# Effect of the duration of bladder overdistention on renal function and morphology in rats

**DOI:** 10.3892/etm.2013.1028

**Published:** 2013-03-26

**Authors:** HONG-ZHOU MENG, MIN CAO, JIAN-JIAN XIANG, XIE-LAI ZHOU, HONG-PING YIN, BAI-YE JIN, ZHAO-DIAN CHEN, XIAO-DONG JIN

**Affiliations:** 1 Department of Urology, The First Affiliated Hospital, Zhejiang University School of Medicine, Hangzhou, Zhejiang 310003;; 2 Medical Research Center, The Second Chengdu Hospital Affiliated to Chongqing Medical University, Chengdu, Sichuan 610031;; 3 Department of Ultrasonography, The First Affiliated Hospital, Zhejiang University School of Medicine, Hangzhou, Zhejiang 310003;; 4 College of Medicine, Hangzhou Normal University, Hangzhou, Zhejiang 310036, P.R. China

**Keywords:** acute urinary retention, overdistention, rat, serum creatinine, terminal deoxynucleotidyl transferase-mediated dUTP nick end labelling, ultrastructure

## Abstract

The aim of this study was to investigate the influence of the duration of bladder overdistention (DOBO) on kidney structure and function in rats. Bladder overdistention was induced in male Sprague-Dawley rats by an infusion of saline. Forty rats were divided into five groups: DOBO 1, 2, 4 or 8 h groups and the control. Renal function was evaluated using serum creatinine (SCr) and blood urea nitrogen (BUN). Apoptotic indices and morphologic changes of the kidney were detected by terminal deoxynucleotidyl transferase-mediated dUTP nick end labelling (TUNEL) staining and transmission electron microscopy (TEM). Compared with the control, rats undergoing 2, 4 or 8 h of overdistention showed significant, time-dependent increases in SCr (12.375 vs. 23.125, 34.375 and 51.500 *μ*mol/l, respectively), BUN (6.980 vs. 18.689, 25.184 and 32.079 mmol/l, respectively), renal size (1.041 vs. 1.472, 1.484 and 1.634 cm^3^, respectively) and renal pelvis separation (0.000 vs. 0.223, 0.320, 0.308 and 0.277 cm, respectively; P<0.01). In the rats, 2, 4 and 8 h of overdistention elicited time-dependent increases in the blood flow rate in the main renal artery (MRA; 44.827 vs. 49.082, 59.688 and 67.123 cm^3^/sec, control vs. DOBO 2, 4 and 8 h), the interlobar renal artery (IRA; 32.095 vs. 39.16 and 51.745 cm^3^/sec, control vs. DOBO 4 and 8 h) and the segmental renal artery (SRA; 21.171 vs. 24.355 and 25.358 cm^3^/sec, control vs. DOBO 4 and 8 h; P<0.01). TUNEL results showed that prolonged overdistention increased the apoptotic index of renal cells significantly (1.15, 1.77, 3.40, 5.34 and 13.91% for control and DOBO 1, 2, 4 and 8 h, respectively; P<0.01) and TEM indicated that prolonged overdistention resulted in ultrastructural injuries of increased severity. DOBO plays a significant role in the functional and structural impairment of the rat kidney. With increasing duration, the hemodynamic changes, cell apoptosis and ultrastructural injuries of the kidney are more evident, all of which may contribute to the increasingly serious impairment of renal function and morphology.

## Introduction

Acute urinary retention (AUR) is a common complication in patients with benign prostatic hyperplasia (BPH) (1,2). Previous studies have shown that >10% of men >70 years old experience at least one episode of AUR over a 5-year period and this risk increases to one-third of men over a 10-year period (3).

Urinary retention induces an increase of intravesical pressure, which may affect renal function and morphology. Chronic urinary retention may impair renal function and lead to terminal kidney failure within a few years (4). Whether AUR results in an impairment of renal function and morphology requires investigation. A previous study observed that AUR affects glomerular and tubular renal function, which results in elevated urinary albumin excretion. Following AUR, glomerular permeability and tubular damage persists in the majority of patients (5). Data concerning renal function and morphology, however, remain scarce for AUR.

In clinical practice, the majority of patients with AUR are treated with catheterization (6). However, the durations of AUR prior to intervention differ markedly among patients due to differences in medical history and pain tolerance. Whether different AUR durations result in different impairments of renal function or morphology remains unclear; little has been published in the literature. In the current study, a rat model was used to investigate the effect of the duration of bladder overdistention (DOBO) on renal function and morphology, which may partly clarify this clinical uncertainty and provide information concerning the mechanisms involved in the impairment.

## Materials and methods

### Animal model

Studies were performed on male Sprague-Dawley rats weighing 200–250 g (n=40). All rats received a standard diet, water *ad libitum* and were housed in a 12 h light/dark cycle. All animal care and experimental protocols were in accordance with the guidelines of Zhejiang University (Hangzhou, China). The 40 rats were allocated to five groups: the sham-operated control and DOBO 1, 2, 4 and 8 h groups (each n=8). Rats were anesthetized with urethane (1.0 g/kg i.p.) and anesthesia was maintained by supplementary injections of the same anesthetic. Once anesthetized, the rats were shaved for kidney Doppler ultrasound. The rat bladder was identified with a low midline abdominal incision. After emptying the bladder, the foreskin was ligated using 3-0 silk thread. A 24-G catheter was inserted into the apex of the bladder dome. The catheter was connected to an infusion pump, then 37°C 0.9% saline was infused (0.1 ml/min) until the total volume reached 1 ml. This was twice the mean bladder capacity of 0.5 ml established in preliminary experiments. The status of overdistention was maintained for 1, 2, 4 and 8 h, respectively. In the rats of the control group, the bladder was exposed and punctured but no saline was infused.

### Doppler ultrasound detection

Kidney ultrasound was applied to all rats 0.5 h after the overdistention was relieved. The kidney length, width and cortex thickness were measured. Renal size was calculated using the following formula: Renal size (cm^3^) = renal length (cm) × renal width (cm) × cortex thickness (cm)/6. At the same time, the thicknesses of the cortex and hydronephrosis levels were measured. Furthermore, Doppler ultrasound with a V4 MHz transducer was used to calculate the resistant index (RI) of the main renal artery (MRA), inter lobular artery (IRA) and segmental renal artery (SRA). The RI was calculated with the following formula: RI = (peak systolic shift − minimum diastolic shift)/peak systolic shift (4).

### Renal function test

Blood samples were collected and stored at 4°C until examination. Serum creatinine (SCr) and blood urea nitrogen (BUN) were measured by the Department of Clinical Chemistry using an enzymatic method.

### Terminal deoxynucleotidyl transferase-mediated dUTP nick end labelling (TUNEL) assay

Apoptosis was detected using the TUNEL assay. Formalin-fixed, paraffin wax-embedded tissue sections were deparaffinized and stained using the TUNEL-avidin-biotin-complex method. Ten high-power (×400) fields were randomly selected in one section. The numbers of apoptotic cells, defined by chromatin condensation or nuclear fragmentation were counted. The apoptotic index was calculated as follows: Apoptotic index = number of positive cells/total number of cells × 100.

### Transmission electron microscopy (TEM)

To observe the ultra-structure of the bladder tissues, sections were stained with uranyl acetate and plumbic citrate and examined using TEM (TECNAI 10, Philips, Amsterdam, The Netherlands).

### Statistical analysis

All results are expressed as the mean ± standard error of the mean (SEM). The statistical significance of the difference between two variables was determined using the unpaired Student’s t-test. One-way ANOVA was used to compare variables among more than two groups. P<0.05 was considered to indicate a statistically significant result.

## Results

### Survival

All rats survived and data were collected, with the exception of one rat in the 8 h overdistention group.

### Doppler ultrasound detection

**Figure 1:**
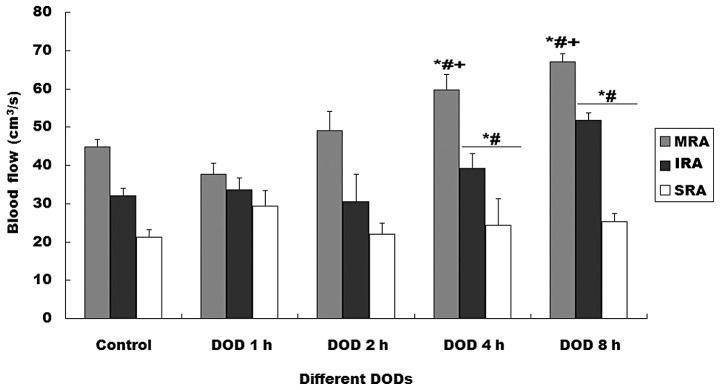
Effect of the duration of bladder overdistention (DOBO) on the blood flow of the renal arteries. Blood flow through the main renal artery (MRA; 44.827 vs. 49.082, 59.688 and 67.123 cm^3^/sec, control vs. DOBO 2, 4 and 8 h), interlobular renal artery (IRA; 32.095 vs. 39.16 and 51.745 cm^3^/sec, control vs. DOBO 4 and 8 h) and segmental renal artery (SRA; 21.171 vs. 24.355 and 25.358 cm^3^/sec, control vs. DOBO 4 and 8 h) increased with distention time (P<0.01). ^*^P<0.01 vs. control group, ^#^P<0.01 vs. 1 h DOBO group, ^+^P<0.01 vs. 2 h DOBO group.

**Figure 2:**
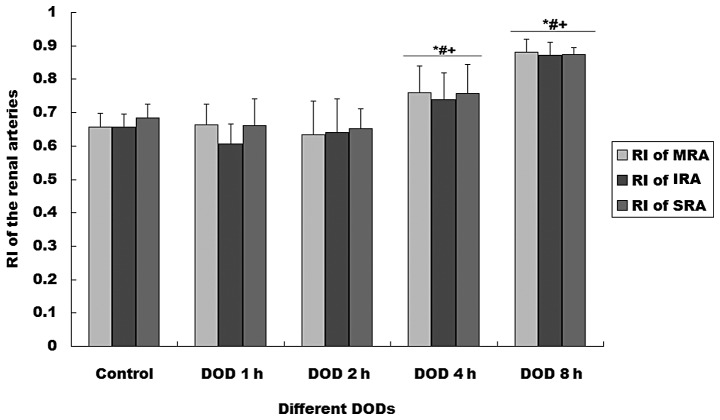
Effect of the duration of bladder overdistention (DOBO) on the resistive index (RI) of the renal arteries. Rats with 4 or 8 h DOBO showed significant increases in the RI of the main renal artery (MRA; 0.657 vs. 0.76 and 0.88), interlobular renal artery (IRA; 0.655 vs. 0.738 and 0.871) and segmental renal artery (SRA; 0.684 vs. 0.757 and 0.873) compared with the control, DOBO 1 and 2 h groups. ^*^P<0.01 vs. control group, ^#^P<0.01 vs. 1 h DOBO group, ^+^P<0.01 vs. 2 h DOBO group.

DOBO had significant effects on renal volume, the degree of separation of the pelvis and cortical thickness (Table I). Compared with the control, the renal size in the rats with 2, 4 or 8 h DOBO was significantly increased (P<0.05). Compared with the DOBO 1 h group, the DOBO 4 and 8 h groups demonstrated significant increases in renal size (P<0.05). A significant difference was also observed between the DOBO 2 h group and the DOBO 4 and 8 h groups (P<0.05). Furthermore, rats with a DOBO of 2, 4 or 8 h showed a significantly higher degree of separation of the pelvis than the controls (P<0.05); significant differences were also observed between the DOBO 1 h group and the 2, 4 and 8 h groups (P<0.05). Cortical thicknesses showed no significant differences among the groups, however. Different overdistention durations had different effects on the blood flow of the renal arteries (Fig. 1). Blood flow in the MRA, IRA and SRA increased with distention time (P<0.01). The RIs of the MRA and SRA were significantly higher in the rats with 4 or 8 h DOBO than in the other groups (P<0.01, Fig. 2).

### Renal function test

Rats with 2, 4 or 8 h DOBO showed significant, time-dependent increases in SCr and BUN levels compared with sham-operated controls (P<0.01, Table II).

### TUNEL assay

**Figure 3:**
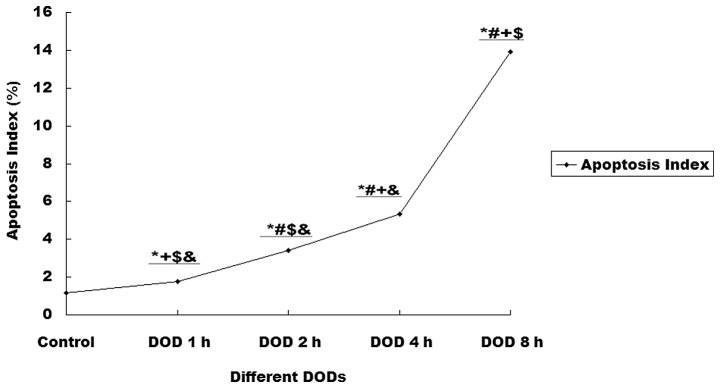
Effect of the duration of bladder overdistention (DOBO) on the apoptotic index of renal cells. Compared with the control, rats with 1, 2, 4 or 8 h DOBO showed a significant increase in apoptotic index (1.15 vs. 1.77, 3.4, 5.34 and 13.91%, respectively; P<0.01) and this effect was time-dependent. ^*^P<0.01 vs. control group, ^#^P<0.01 vs. 1 h DOBO group, ^+^P<0.01 vs. 2 h DOBO group, ^$^P<0.01 vs. 4 h DOBO group, ^&^P<0.01 vs. 8 h DOBO group.

**Figure 4:**
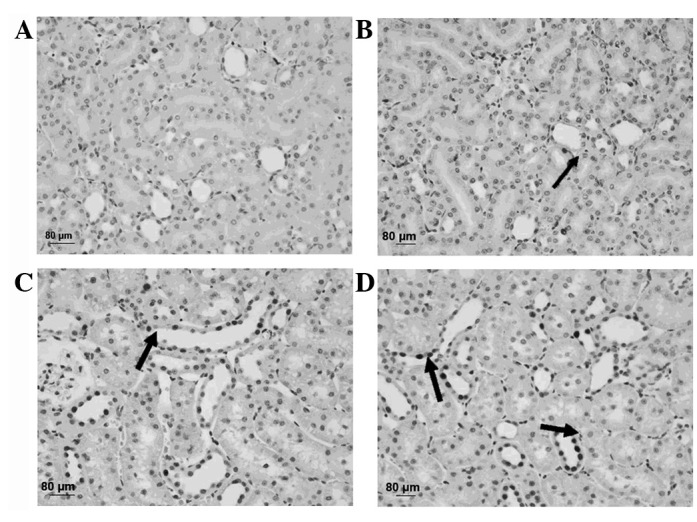
Representative microscopic findings indicating the effect of the duration of duration of bladder overdistention (DOBO) on renal ultra-structure. (A) Control group (magnification, ×200). (B) 1 h DOBO group (magnification, ×200). (C) 4 h DOBO group (magnification, ×200). (D) 8 h DOBO group (magnification, ×200). The number of apoptotic cells (arrows) increased with duration time.

Increasing DOBO also promoted apoptosis within renal tissues. Compared with controls, the rats with 4 or 8 h DOBO showed significant increases in the apoptotic index (P<0.01). This effect was time-dependent (Figs. 3 and 4).

### TEM

**Figure 5:**
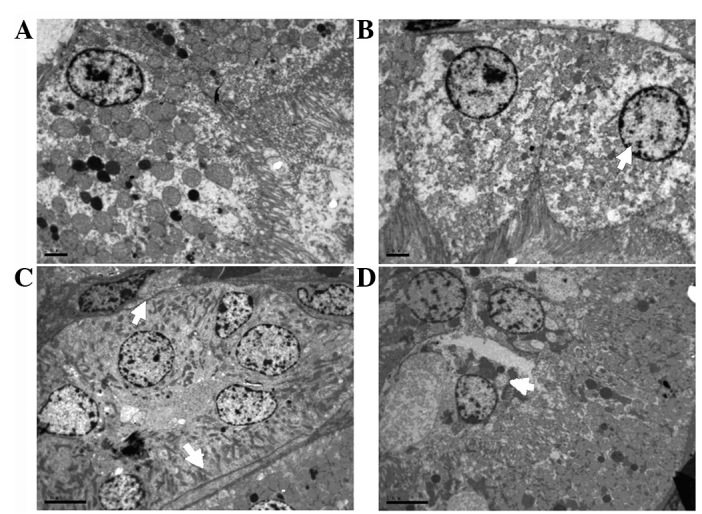
Representative microscopic findings indicating the effect of the duration of bladder overdistention (DOBO) on renal ultrastructure. (A) Control group (magnification, ×2,550). (B) 1 h DOBO group (magnification, ×2,550). (C) 4 h DOBO group (magnification, ×1,850). (D) 8 h DOBO group (magnification, ×1,850). Electron microscopy revealed that an increase in the DOBO led to increasingly aggravated cellular and tissue injury. Evidence of injury included mitochondrial breakdown, tubular vacuolization and dilation, mesangial proliferation, necrosis, podocyte swelling and inosculation and clearly visible slit pores (arrows).

Electron microscopy revealed that increasing DOBO led to increasingly aggravated cellular and tissue injury. Evidence of injury included mitochondrial breakdown, tubular vacuolization and dilation, mesangial proliferation, necrosis, podocyte swelling and inosculation and slit pores were clearly visible. These injuries became more pronounced as the DOBO increased (Fig. 5).

## Discussion

It is well known that BPH is a progressive disease, with risk of urinary retention and renal insufficiency (5). Renal impairment caused by BPH is usually a chronic disorder, which takes several years, even decades to develop (9). In the current study, it was identified that rats with bladder overdistention had significant, time-dependent reductions in SCr and BUN levels.

Renal hemodynamic alterations and the consequences of progression to irreversible renal injury following unilateral ureteral obstruction (UUO) have been studied in detail (6). In animals with UUO, both renal blood flow (RBF) and glomerular filtration rate (GFR) decrease and remain depressed without intervention (7). The degree of damage to renal function depends on the duration of UUO and the species being examined (1,12,13). In the current study, the blood flow rate was observed to increase in the renal arteries during overdistention in a time-dependent manner, which was associated with increasingly severe hydronephrosis and increasing renal size. Kidney length is traditionally used to predict kidney size; however, length alone is an unreliable predictor of kidney size and the number of functional nephrons (8), a limitation noted in both human and animal studies (9). In the present study, renal size and hydronephrosis were used as indices of renal morphology. During overdistention, intrapelvic pressure increases until ureteral regurgitation occurs, resulting in hydronephrosis and a decrease in renal perfusion without cellular injury (10). In compensation for this, the renal arteries became constricted, driving an increase in the blood flow rate. Accordingly, the affected kidney will show reduced ability to concentrate urine and, if tubular and glomerular damage are allowed to progress, renal failure may follow (11).

In the present study, it was also observed that overdistention aggravated ultrastructural changes and increased the number of apoptotic cells. Early morphological changes in response to overdistention include the formation of blebs in the apical membranes of proximal tubule cells, as well as loss of the brush border (12,13). Proximal tubule cells lost their polarity and the integrity of their tight junctions was disrupted, which may have been consequences of alterations in actinomycin-D microtubule cytoskeleton networks (14). In addition, Na^+^/K^+^-ATPase redistributes from the basolateral to the apical membrane, contributing to a decrease in Na^+^ and Na^+^-coupled vectorial transport. Integrins are redistributed to the apical surface (15), and live and dead cells slough off into the tubular lumen, contributing to the formation of urinary casts. The casts cause increased intratubular pressure and a decrease in GFR. Loss of the epithelial cell barrier and tight junctions between viable cells may result in back-leakage of the glomerular filtrate, further reducing the effective GFR.

It was postulated that renal injury due to ischemia may involve the generation of reactive oxygen species, production of inflammatory cytokines, inflammatory cell infiltration and the production of fibronectin and collagen, all of which contribute to interstitial fibrosis and renal microvascular injury. In the present study, the main ultrastructural changes were mitochondrial breakdown and disorganized podocytes; these and all other negative ultrastructural changes were further exacerbated by increasing durations of overdistention.

Based on these results and those reported previously, bladder overdistention affects not only the structure and function of the bladder but also of the kidney. Thus, more attention should be paid to the effects of AUR on the upper urinary tract. Clinical practice should be expanded to include monitoring of renal function in patients with AUR, especially in cases of prolonged AUR. These results indicate that the bladder should be decompressed as quickly as possible and the duration of AUR should be shortened to protect renal function in the treatment of AUR. Certainly, overdistention of the bladder is not the same as AUR and clinical circumstances are more complicated than animal experiments. Other factors such as comorbidity, age and physical conditions also contribute to the severity of impairment and the recovery of renal function following AUR.

In the present study, there was no recovery of renal function after the overdistention was relieved. How the duration of overdistention affects the recovery of renal insufficiency should be studied further.

DOBO plays an important role in functional and structural impairment of the kidney. Different overdistention durations lead to different severities of impairment of the rat kidney. With increasing duration, the hemodynamic changes, cell apoptosis and ultrastructural injuries of the kidney are more evident, all of which may contribute to more serious impairment of renal function and morphology.

## Figures and Tables

**Table I:** Effect of the duration of bladder overdistention (DOBO) on renal size, degree of separation of the renal pelvis and the thickness of the renal cortex.

Group	Renal size (cm^3^)	Separation of the pelvis (cm)	Thickness of the renal cortex (cm)
Control (n=8)	1.041±0.159	0.000±0.000	0.263±0.037
1 h DOBO (n=8)	1.146±0.162	0.223±0.059^a^	0.259±0.058
2 h DOBO (n=8)	1.472±0.145^a^	0.320±0.029^a,b^	0.276±0.032
4 h DOBO (n=8)	1.484±0.193^a–c^	0.308±0.044^a–c^	0.251±0.052
8 h DOBO (n=7)	1.634±0.155^a–c^	0.277±0.028^a–c^	0.256±0.035

Data are reported as the mean ± standard error of the mean (SEM).

a P<0.05 vs. control group,

b P<0.05 vs. 1 h DOBO group,

c P<0.05 vs. 2 h DOBO group.

**Table II:** Effect of the duration of bladder overdistention (DOBO) on serum creatinine (SCr) and blood urea nitrogen (BUN) levels.

Group	SCr (*μ*mol/l)	BUN (mmol/l)
Control (n=8)	12.375±1.302	6.980±1.072
1 h DOBO (n=8)	18.250±1.669	11.481±1.474^a^
2 h DOBO (n=8)	23.125±2.532^a,b^	18.689±1.100^a,b^
4 h DOBO (n=8)	34.375±2.200^a–c^	25.184±1.760^a–c^
8 h DOBO (n=7)	51.500±3.423^a–d^	32.079±5.592^a–d^

Data are reported as the mean ± standard error of the mean (SEM).

a P<0.05 vs. control group,

b P<0.05 vs. 1 h DOBO group,

c P<0.05 vs. 2 h DOBO group,

d P<0.05 vs. 4 h DOBO group.
